# Validation and factor analysis of the parental bonding instrument in Japanese pregnant women

**DOI:** 10.1038/s41598-021-93146-3

**Published:** 2021-07-02

**Authors:** Maya Sato, Takashi Okada, Mako Morikawa, Yukako Nakamura, Aya Yamauchi, Masahiko Ando, Norio Ozaki

**Affiliations:** 1 Department of Psychiatry, Nagoya University Graduate School of Medicine, 65 Tsurumai-cho, Showa-ku, Nagoya, Aichi 466-8550 Japan; 2 Department of Medical Technique, Nagoya University Hospital, 65 Tsurumai-cho, Showa-ku, Nagoya, Aichi 466-8550 Japan; 3 Center of Advanced Medicine and Clinical Research, Nagoya University Hospital, 65 Tsurumai-cho, Showa-ku, Nagoya, Aichi 466-8550 Japan

**Keywords:** Depression, Disease prevention

## Abstract

The Parental Bonding Instrument (PBI) evaluates parental attitudes derived from an individual’s childhood experiences with their parents. The factor structure of the PBI differs depending on variables such as psychosocial factors including culture, race, sex, and psychological and social conditions of participants. Although previous studies of the relationships between perinatal depression and parenting experiences have used the factor structures of the PBI from the general population, it is unclear whether the same factor structures are appropriate in the highly variable perinatal period. In this study, complete responses to the PBI and the Edinburgh Postnatal Depression Scale (EPDS) were received from 932 primiparas at 25 weeks of gestation and at 1 month postpartum. An exploratory factor analysis was performed on half of the responses, and it was confirmed that the three factors were care, interference, and autonomy. Confirmatory factor analysis of the remaining half of the answers showed comprehensible fitness. Each factor showed a high degree of internal consistency, and each factor of the PBI correlated with the EPDS, indicating construct validity. The reliability and validity of the PBI in perinatal Japanese women were confirmed, and it was found that the PBI had a three-factor structure.

## Introduction

Individuals’ childhood experiences with their parents affect their personal, psychological, and social development in adulthood^1^. It has been suggested that childhood maltreatment is a risk factor for detrimental effects on mental health that may extend to adulthood^2,3^. Therefore, Roe et al. and Schaefer developed a questionnaire to measure the effects of parental behavior on children^4,5^. They then analyzed the answers to questions about parent’s behavior towards themselves when they were children, targeting both children and adults^4^. The results showed that parents’ behaviors were composed of two or three factors. Schaefer analyzed the responses of independent samples of children and adults in a similar study^5^.

Based on the findings of the studies reviewed, Parker et al. considered that the parental contribution to bonding may be influenced by two principal source variables. To assist in the definition of the two principal dimensions, they asked Australian students and adolescents to respond to a questionnaire including items suggesting parental behaviors and attitudes, including care, and they then performed a factor analysis of the results. They reported that parental attitudes consisted of two factors, care and overprotection, and created the Parental Bonding Instrument (PBI) questionnaire to assess parental attitudes based on an individual’s experiences of being parented or memories of parenting during childhood. In addition, they showed that the PBI was found to be reliable and valid, and the scales appear to be acceptable^6^.

Many studies have examined the relationship between the PBI and mental disorders to test the hypothesis that parental attitudes are also related to personality development and the development of mental illness. For example, low levels of care and high levels of overprotection have been reported to be associated with depressive symptoms^6,7^.

However, other studies have identified that parents’ attitudes toward parenting may differ depending on their background, including factors such as culture, religion, customs, race, sex, personality, mental illness, and social status^6,8^. For example, a study by Cubis et al., which was carried out ten years after Parker’s study and was similarly carried out in Australia, reported a care factor and two protection factors from a community sample of adolescents, as well as a sex difference that was not found in Parker’s study^9^. A study of students in the United Kingdom and the United States in the same time period as the study by Cubis et al. also reported that three factors (care, denial of psychological autonomy, and encouragement of behavioral freedom) were more satisfactory than two factors^10^.

In Japan, Kitamura et al.^11^ administered the PBI to children and their parents and reported a two-factor structure consisting of care and overprotection. However, later studies examining the factor structure of the PBI in working adults supported the three-factor structures^12,13^. Elsewhere, in a study that examined the factor structure solely among parents with children (aged 6–14 years), Uji et al.^14^ proposed a four-factor structure of care, indifference, overprotection, and autonomy and also identified sex differences.

In several previous studies of factor analysis of the PBI in women, although mothers with children in kindergarten demonstrated four factors, i.e., care, indifference, overprotection, and autonomy^15^, those aged 20–35 years exhibited two factors, i.e., care and overprotection^16^, and women three days after giving birth were found to exhibit three factors, i.e., affect, overprotection, and restraint^17^. From these results, it was suggested that, even if a study were conducted for the same region and the same sex, there was a possibility that the factor structure of the PBI might differ depending on participants’ situations and backgrounds at that time.

Perinatal women undergo significant changes in their physical, mental, and social situations, increasing the risk of developing mental illnesses, especially depressive symptoms; thus, the prevention and identification of risk factors in this regard is a pressing research topic. For this reason, some studies have focused on perinatal women to determine whether participants’ childhood experiences of being parented can be a risk factor for depression. Boyce et al.^18^ reported that paternal overprotection and low maternal care were risk factors for postpartum depression. McMahon et al.^19^ also reported that low maternal care was a risk factor for postpartum depression. However, these studies used two factors derived from the general population: care and overprotection.

When examining the relationship between mental health during pregnancy and postpartum, it is possible to verify the relationship between psychosocial factors in perinatal women and perinatal depression by using a PBI factor structure specifically for perinatal women and by examining the relationship between these factors and depression. However, there has been only one factor analysis of the PBI in perinatal women that was reported 30 years ago in Spain, at only one point on the third postpartum day, and the number of women (205) was small^17^. Furthermore, previous studies have examined the association with postpartum depression using the results of the factor structure of the PBI in postnatal women, without confirming whether the PBI throughout the prenatal and postpartum periods was applicable, and we believe that needs to be tested.

In this study, the reliability, validity, and factor structure of the PBI were investigated during pregnancy and in the postpartum period in a prospective cohort of perinatal women.

## Results

### Descriptive statistics

A total of 1362 perinatal women agreed to participate in the study between April 2006 and May 2020. The data of 932 participants (response rate 68.4%) who completed both the EPDS and PBI questionnaires at 25 weeks of gestation (T1) and at 1 month postpartum (T2) were used. Their mean age was 32.67 (standard deviation (SD) =  ± 4.69; 20–45) years. The 932 participants were randomly divided into two groups; Group 1 (mean age ± SD, 32.60 ± 4.740 years; mean EPDS scores ± SD at T1, 4.98 ± 4.62; at T2, 5.52 ± 4.77) and Group 2 (mean age, 32.73 ± 4.65 years; mean EPDS scores at T1, 5.09 ± 4.85; at T2, 5.13 ± 4.82) comprised 466 and 466 perinatal women, respectively.

### Factor analysis of the parental bonding instrument

Prior to the factor analysis, the data distributions were checked for normality (Tables 1, 2). Most of the items were confirmed to be normally distributed (skewness < 2.0, kurtosis < 7.0)^20^. Some of the items were slightly above the upper limit of skewness, but based on the results of past factor analyses and the content of the items, the items were considered to be important within the questionnaire. The validity of the factor analysis was examined by the Kaiser–Meyer–Olkin (KMO) measure of sampling adequacy. The KMO measures of sampling adequacy were: paternal PBI at T1 0.945, paternal PBI at T2 0.943, maternal PBI at T1 0.946, and maternal PBI at T2 0.953. The KMO measure of sampling adequacy at each time point had a reasonable value for factor analysis. The number of factors was examined from the scree plot, and the three-factor structure was considered in exploratory factor analyses (EFAs).

**Table 1:** Means, SD, skewness, and kurtosis in this study (Fathers).

PBI items	T1			T2		
	M (SD)	Skewness	Kurtosis	M (SD)	Skewness	Kurtosis
1. Spoke to me with a warm and friendly voice	2.03 (0.95)	− 0.60	− 0.69	2.11 (0.92)	− 0.68	− 0.54
2. Did not help me as much as I needed	1.84 (0.96)	− 0.45	− 0.75	1.86 (1.01)	− 0.46	− 0.91
3. Let me do those things I liked doing	0.62 (0.86)	1.28	0.75	0.64 (0.80)	1.12	0.61
4. Seemed emotionally cold to me	2.45 (0.80)	− 1.48	1.64	2.43 (0.82)	− 1.35	1.02
5. Appeared to understand my problems and worries	1.60 (0.96)	− 0.067	− 0.94	1.63 (0.95)	− 0.039	− 0.96
6. Was affectionate to me	2.27 (0.87)	− 1.00	0.14	2.30 (0.85)	− 1.09	0.44
7. Liked me to make my own decisions	0.87 (0.95)	0.81	− 0.36	0.79 (0.88)	0.83	− 0.23
8. Did not want me to grow up	0.69 (0.77)	1.05	0.85	0.62 (0.78)	1.16	0.83
9. Tried to control everything I did	0.51 (0.82)	1.59	1.74	0.55 (0.84)	1.39	0.91
10. Invaded my privacy	0.50 (0.78)	1.56	1.80	0.53 (0.81)	1.39	1.03
11. Enjoyed talking things over with me	1.93 (1.01)	− 0.50	− 0.91	2.02 (0.97)	− 0.61	− 0.69
12. Frequently smiled at me	1.88 (1.00)	− 0.40	− 0.98	1.94 (0.98)	− 0.47	− 0.88
13. Tended to baby me	0.93 (0.27)	0.27	− 0.75	1.27 (0.93)	0.35	− 0.69
14. Did not seem to understand what I needed	2.08 (0.91)	− 0.77	− 0.19	2.14 (0.89)	− 0.78	− 0.27
15. Let me decide things for myself	0.97 (0.90)	0.66	− 0.31	0.91 (0.87)	0.58	− 0.55
16. Made me feel I wasn’t wanted	2.69 (0.70)	− 2.58	6.39	2.66 (0.74)	− 2.22	4.08
17. Could make me feel better when I was upset	1.44 (1.01)	0.12	− 1.07	1.48 (1.01)	0.066	− 1.09
18. Did not talk with me very much	1.84 (1.02)	− 0.36	− 1.03	1.94 (1.03)	− 0.57	− 1.03
19. Tried to make me dependent on her/him	0.57 (0.67)	1.02	0.89	0.63 (0.78)	1.20	1.05
20. Felt I could not look after myself unless	0.27 (0.54)	2.34	6.59	0.26 (0.54)	2.13	4.45
21. Gave me as much freedom as I wanted	0.81 (0.88)	0.91	0.060	0.79 (0.87)	0.84	− 0.20
22. Let me go out as often as I wanted	1.17 (1.00)	0.33	− 1.04	1.06 (1.00)	0.51	− 0.86
23. Was overprotective of me	1.19 (0.97)	0.34	− 0.89	1.15 (095)	0.37	− 0.82
24. Did not praise me	2.03 (0.96)	− 0.72	− 0.46	2.06 (0.96)	− 0.68	− 0.60
25. Let me dress in any way I pleased	0.68 (0.87)	1.13	0.40	0.62 (0.84)	1.28	0.84

PBI: Parental Bonding Instrument.

**Table 2:** Means, SD, skewness, and kurtosis in this study (Mothers).

PBI items	T1			T2		
	M (SD)	Skewness	Kurtosis	M (SD)	Skewness	Kurtosis
1. Spoke to me with a warm and friendly voice	2.48 (0.74)	− 1.29	0.95	2.56 (0.67)	− 1.44	1.63
2. Did not help me as much as I needed	2.30 (0.89)	− 1.10	0.26	2.34 (0.85)	− 1.13	0.46
3. Let me do those things I liked doing	0.64 (0.74)	0.93	0.28	0.65 (0.78)	1.05	0.55
4. Seemed emotionally cold to me	2.62 (0.68)	− 1.92	3.33	2.62 (0.69)	− 1.83	2.92
5. Appeared to understand my problems and worries	2.18 (0.91)	− 0.81	− 0.33	2.22 (0.82)	− 0.76	− 1.89
6. Was affectionate to me	2.48 (0.74)	− 1.35	1.32	2.51 (0.68)	− 1.22	0.85
7. Liked me to make my own decisions	0.80 (0.88)	0.80	− 0.31	0.78 (0.86)	0.86	− 0.11
8. Did not want me to grow up	0.66 (0.85)	1.18	0.63	0.61 (0.81)	1.14	0.43
9. Tried to control everything I did	0.82 (0.93)	0.85	− 0.34	0.83 (0.93)	0.76	− 0.54
10. Invaded my privacy	0.70 (0.87)	1.08	0.31	0.79 (0.90)	0.85	− 0.26
11. Enjoyed talking things over with me	2.53 (0.75)	− 1.55	1.77	2.57 (0.69)	− 1.54	1.79
12. Frequently smiled at me	2.44 (0.79)	− 1.21	0.51	2.50 (0.75)	− 1.38	1.14
13. Tended to baby me	1.26 (0.99)	0.31	− 0.93	1.25 (0.98)	0.19	− 1.03
14. Did not seem to understand what I needed	2.22 (0.83)	− 0.88	0.18	2.26 (0.82)	− 0.91	0.13
15. Let me decide things for myself	0.86 (0.83)	0.67	− 0.25	0.86 (0.84)	0.60	− 0.48
16. Made me feel I wasn’t wanted	2.65 (0.76)	− 2.28	4.38	2.68 (0.73)	− 2.37	4.75
17. Could make me feel better when I was upset	2.21 (0.92)	− 0.91	− 0.18	2.27 (0.89)	− 1.02	0.081
18. Did not talk with me very much	2.79 (0.65)	− 2.30	4.99	2.70 (0.64)	− 2.19	4.38
19. Tried to make me dependent on her/him	0.68 (0.85)	1.13	0.50	0.76 (0.89)	0.91	− 0.14
20. Felt I could not look after myself unless	0.48 (0.77)	1.60	1.94	0.51 (0.80)	1.48	1.32
21. Gave me as much freedom as I wanted	0.80 (0.87)	0.87	− 0.13	0.84 (0.88)	0.70	− 0.45
22. Let me go out as often as I wanted	118 (0.99)	0.37	− 0.93	1.07 (0.96)	0.41	− 0.90
23. Was overprotective of me	1.43 (1.05)	0.043	− 1.21	1.38 (1.05)	0.075	− 1.22
24. Did not praise me	2.10 (0.86)	− 1.03	0.15	2.33 (0.86)	− 1.08	0.22
25. Let me dress in any way I pleased	0.62 (0.78)	1.01	0.19	0.66 (0.83)	1.01	0.069

PBI: Parental Bonding Instrument.

For the purpose of factor analyses, only data from the 932 subjects who answered all the PBI items and EPDS items at T1 and T2 were used. The subjects were randomly divided into two groups, and an EFA of the 25 items of the PBI was conducted in Group 1. For the paternal PBI at T1, four items (7, 10, 14, 23) had a factor loading > 0.30 for multiple factors. For the maternal PBI at T1, four items (7, 14, 16, 23) had a factor loading > 0.30 for multiple factors. For both the paternal and maternal PBIs at T2, three items (7, 14, 23) had a factor loading > 0.30 for multiple factors. An EFA was performed again when each item was deleted, and it was confirmed that the factor structure was stable in all cases. Since EFA yielded multiple models of the factor structure, a confirmatory factor analysis (CFA) using Group 2 was performed for each of the models, and the goodness of fit was confirmed.

For both the paternal and maternal PBIs, the model excluding three items (7, Liked me to make my own decisions; 14, Did not seem to understand what I needed or wanted; 23, Was overprotective of me) had the best fit. Both paternal and maternal PBIs suggested a three-factor structure (Tables 3, 4).

**Table 3:** Factor matrix of the Parental Bonding Instrument after rotation (Fathers).

	T1			T2		
	Factor 1	Factor 2	Factor 3	Factor 1	Factor 2	Factor 3
**Care**						
1. Spoke to me with a warm and friendly voice	**0.773**	0.180	− 0.173	**0.773**	0.181	− 0.222
2. Did not help me as much as I needed	**0.758**	− 0.071	0.184	**0.738**	− 0.136	0.122
4. Seemed emotionally cold to me	**0.859**	− 0.124	0.188	**0.888**	− 0.150	0.244
5. Appeared to understand my problems and worries	**0.648**	0.021	− 0.128	**0.605**	− 0.066	− 0.126
6. Was affectionate to me	**0.762**	0.082	− 0.151	**0.747**	0.076	− 0.202
11. Enjoyed talking things over with me	**0.816**	0.282	− 0.151	**0.813**	0.292	− 0.191
12. Frequently smiled at me	**0.847**	0.220	− 0.109	**0.851**	0.262	− 0.171
14. Did not seem to understand what I needed	**0.649**	**-0.326**	0.017	**0.610**	**-0.472**	0.067
16. Made me feel I wasn’t wanted	**0.696**	− 0.141	0.131	**0.669**	− 0.275	0.234
17. Could make me feel better when I was upset	**0.681**	0.115	− 0.146	**0.667**	0.159	− 0.156
18. Did not talk with me very much	**0.831**	0.106	0.115	**0.795**	0.147	0.065
24. Did not praise me	**0.825**	− 0.154	0.155	**0.748**	− 0.125	0.049
**Interference**						
8. Did not want me to grow up	− 0.249	**0.459**	0.081	− 0.280	**0.413**	0.065
9. Tried to control everything I did	− 0.098	**0.564**	0.194	− 0.101	**0.642**	0.184
10. Invaded my privacy	**-0.326**	**0.573**	− 0.047	− 0.172	**0.615**	0.090
13. Tended to baby me	0.137	**0.559**	0.035	0.176	**0.599**	0.036
19. Tried to make me dependent on her/him	0.103	**0.434**	0.097	0.196	**0.357**	0.141
20. Felt I could not look after myself unless	0.029	**0.486**	0.102	0.039	**0.479**	0.100
23. Was overprotective of me	**0.330**	**0.452**	0.047	**0.493**	**0.351**	0.277
**Autonomy**						
3. Let me do those things I liked doing	− 0.076	− 0.010	**0.776**	− 0.119	0.182	**0.595**
7. Liked me to make my own decisions	**-0.414**	0.115	**0.409**	**-0.307**	0.183	**0.448**
15. Let me decide things for myself	− 0.172	0.163	**0.535**	− 0.102	0.162	**0.611**
21. Gave me as much freedom as I wanted	0.080	0.136	**0.876**	0.047	0.254	**0.768**
22. Let me go out as often as I wanted	0.232	0.160	**0.727**	0.166	0.136	**0.709**
25. Let me dress in any way I pleased	− 0.256	0.066	**0.343**	− 0.176	0.015	**0.350**

Factors that satisfy the factor loading > 0.3 after factor analysis are shown in bold.

T1: at 25 weeks of gestation; T2: at 1 month postpartum.

**Table 4:** Factor matrix of the Parental Bonding Instrument after rotation (Mothers).

	T1			T2		
	Factor 1	Factor 2	Factor 3	Factor 1	Factor 2	Factor 3
**Care**						
1. Spoke to me with a warm and friendly voice	**0.815**	0.203	− 0.174	**0.826**	0.133	− 0.097
2. Did not help me as much as I needed	**0.607**	− 0.058	0.114	**0.652**	− 0.065	0.090
4. Seemed emotionally cold to me	**0.871**	− 0.103	0.168	**0.839**	− 0.074	0.096
5. Appeared to understand my problems and worries	**0.704**	− 0.057	− 0.066	**0.741**	− 0.035	− 0.036
6. Was affectionate to me	**0.750**	0.157	− 0.235	**0.769**	0.131	− 0.187
11. Enjoyed talking things over with me	**0.854**	0.168	− 0.030	**0.866**	0.202	− 0.040
12. Frequently smiled at me	**0.919**	0.234	− 0.087	**0.920**	0.192	− 0.024
14. Did not seem to understand what I needed	**0.403**	**-0.339**	− 0.141	**0.456**	**-0.330**	− 0.141
16. Made me feel I wasn’t wanted	**0.676**	**-0.316**	0.290	**0.686**	− 0.209	0.196
17. Could make me feel better when I was upset	**0.762**	− 0.019	− 0.018	**0.812**	0.020	0.038
18. Did not talk with me very much	**0.718**	0.026	0.067	**0.843**	0.070	0.098
24. Did not praise me	**0.792**	0.031	− 0.015	**0.740**	− 0.040	0.048
**Interference**						
8. Did not want me to grow up	− 0.295	**0.470**	− 0.023	− 0.239	**0.389**	0.141
9. Tried to control everything I did	− 0.176	**0.569**	0.155	− 0.123	**0.560**	0.223
10. Invaded my privacy	− 0.234	**0.663**	− 0.047	− 0.222	**0.525**	0.085
13. Tended to baby me	0.037	**0.651**	0.007	0.002	**0.723**	− 0.039
19. Tried to make me dependent on her/him	0.208	**0.476**	0.132	0.173	**0.716**	− 0.072
20. Felt I could not look after myself unless	0.007	**0.480**	0.121	− 0.040	**0.749**	− 0.132
23. Was overprotective of me	**0.338**	**0.505**	0.147	**0.303**	**0.623**	0.092
**Autonomy**						
3. Let me do those things I liked doing	− 0.135	0.003	**0.719**	− 0.045	− 0.072	**0.836**
7. Liked me to make my own decisions	**-0.344**	0.127	**0.382**	**-0.304**	0.150	**0.408**
15. Let me decide things for myself	− 0.051	0.149	**0.623**	− 0.072	0.220	**0.570**
21. Gave me as much freedom as I wanted	0.094	0.147	**0.844**	0.063	− 0.010	**0.939**
22. Let me go out as often as I wanted	0.172	0.092	**0.729**	0.202	− 0.088	**0.914**
25. Let me dress in any way I pleased	− 0.178	0.075	**0.418**	− 0.060	0.060	**0.492**

Factors that satisfy the factor loading > 0.3 after factor analysis are shown in bold.

T1: at 25 weeks of gestation; T2: at 1 month postpartum.

The first factor was loaded by 11 items of the PBI (1, 2, 4, 5, 6, 11, 12, 16, 17, 18, and 24); this factor was named “care.” The second factor was loaded by six items of the PBI (8, 9, 10, 13, 19, and 20); this factor was named “interference.” The third factor was loaded by five items in the PBI (3, 15, 21, 22, and 25); this factor was named “autonomy.”

The model derived from the EFA showed suitable fit with the data (for the paternal PBI: Chi-squared (χ^2^)/degrees of freedom (*df*) 2.378, comparative fitness index (CFI) 0.951, standardized root mean square residual (SRMR) 0.0682, root mean square error of approximation (RMSEA) 0.054, and Akaike’s information criterion (AIC) 579.039 at T1, with χ^2^/*df* 2.390, CFI 0.954, SRMR 0.0602, RMSEA 0.055, and AIC 582.123 at T2; for the maternal PBI: χ^2^/*df* 2.263, CFI 0.960, SRMR 0.0607, RMSEA 0.052, and AIC 557.195 at T1, with χ^2^/*df* 2.564, CFI 0.958, SRMR 0.0625, RMSEA 0.058, and AIC 614.784 at T2.) (Table 5).

**Table 5:** Confirmatory factor analysis of the Parental Bonding Instrument items over the two time points in Group 2.

PBI item	Paternal		Maternal	
	T1	T2	T1	T2
**Care**				
1	0.81	0.85	0.80	0.80
2	0.68	0.67	0.74	0.75
4	0.77	0.73	0.81	0.80
5	0.78	0.74	0.73	0.80
6	0.78	0.81	0.80	0.82
11	0.78	0.78	0.78	0.78
12	0.82	0.86	0.83	0.79
16	0.54	0.53	0.63	0.65
17	0.72	0.74	0.81	0.81
18	0.70	0.73	0.75	0.71
24	0.77	0.75	0.72	0.70
**Interference**				
8	0.61	0.68	0.64	0.70
9	0.81	0.82	0.82	0.83
10	0.77	0.79	0.74	0.73
13	0.45	0.50	0.57	0.64
19	0.37	0.45	0.40	0.57
20	0.50	0.62	0.49	0.56
**Autonomy**				
3	0.69	0.71	0.79	0.82
15	0.76	0.75	0.79	0.77
21	0.81	0.87	0.85	0.88
22	0.53	0.61	0.53	0.63
25	0.44	0.49	0.57	0.62
Covariance between Care and Interference	− 0.47	− 0.49	− 0.58	− 0.59
Covariance between Care and Autonomy	− 0.60	− 0.54	− 0.63	− 0.61
Covariance between Interference and Autonomy	0.73	0.79	0.82	0.84
CMIN/*df*	2.378	2.390	2.263	2.564
CFI	0.951	0.954	0.960	0.955
RMSEA	0.054	0.055	0.052	0.058
SRMR	0.0682	0.0602	0.0607	0.0625
AIC	579.039	582.123	557.195	614.784

T1: at 25 weeks of gestation; T2: at 1 month postpartum; χ^2^: Chi-squared; *df*: Degrees of freedom; CFI: Comparative fitness index; SRMR: Standardized root mean square residual; RMSEA: Root mean square error of approximation; AIC: Akaike’s information criterion.

To examine the stability of the factor structure of the PBI, a series of CFAs using all periods from Group 2 were conducted. The path values and accompanying fit indices are shown in Table 4. Examination of CMIN/*df*, CFI, SRMR, and RMSEA showed the current model to meet suitable fit criteria for all periods.

The three factors were moderately correlated with each other (Table 6).

**Table 6:** Spearman’s correlation coefficients of the Parental Bonding Instrument subscale scores and the Edinburgh Postnatal Depression Scale, n = 466.

			T1			T2			T1			T2			EPDS score	
			Father						Mother							
			Care	Interference	Autonomy	Care	Interference	Autonomy	Care	Interference	Autonomy	Care	Interference	Autonomy	T1	T2
T1	Father	Care		− 0.366**	− 0.552**	0.885**	− 0.292**	− 0.479**	0.490**	− 0.307**	− 0.269**	0.459**	− 0.260**	− 0.230**	− 0.264**	
		Interference			0.588**	− 0.336**	0.708**	0.491**	− 0.374**	0.483**	0.395**	− 0.317**	0.392**	0.305**	0.261**	
		Autonomy				− 0.499**	0.515**	0.778**	− 0.401**	0.381**	0.596**	− 0.362**	0.361**	0.481**	0.173**	
T2		Care					− 0.335**	− 0.507**	0.458**	− 0.311**	− 0.256**	0.519**	− 0.305**	− 0.261**	− 0.228**	
		Interference						− 0.546**	− 0.313**	0.405**	0.357**	− 0.341**	0.507**	0.356**	0.164**	
		Autonomy							− 0.363**	0.333**	0.546**	− 0.381**	0.374**	0.597**	0.123**	
T1	Mother	Care								− 0.459**	− 0.521**	0.827**	− 0.394**	− 0.423**		− 0.209**
		Interference									0.570**	− 0.408**	0.746**	0.546**		0.211**
		Autonomy										− 0.477**	0.524**	0.741**		0.104*
T2		Care											− 0.437**	− 0.501**		− 0.214**
		Interference												0.602**		0.174**
		Autonomy														0.155**

PBI: Parental Bonding Instrument; EPDS: Edinburgh Postnatal Depression Scale,

T1: at early pregnancy before week 25; T2: at 1 month postpartum.

*p < 0.05; **p < 0.01.

### Reliability of the parental bonding instrument

Group 1 was used to confirm reliability. Cronbach’s alpha values were: Care 0.937, Interference 0.756, Autonomy 0.847 for the paternal PBI at T1; Care 0.937, Interference 0.761, Autonomy 0.835 for the paternal PBI at T2; Care 0.934, Interference 0.801, Autonomy 0.848 for the maternal PBI at T1; and Care 0.937, Interference 0.831, Autonomy 0.864 for the maternal PBI at T2. Split-half values were: Care 0.947, Interference 0.654, Autonomy 0.814 for the paternal PBI at T1; Care 0.946, Interference 0.776, Autonomy 0.784 for the paternal PBI at T2; Care 0.950, Interference 0.714, Autonomy 0.816 for the maternal PBI at T1; and Care 0.944, Interference 0.808, Autonomy 0.853 for the maternal PBI at T2. McDonald’s omega values were: Care 0.937, Interference 0.751, Autonomy 0.845 for the paternal PBI at T1; Care 0.936, Interference 0.760, Autonomy 0.835 for the paternal PBI at T2; Care 0.933, Interference 0.795, Autonomy 0.848 for the maternal PBI at T1; and Care 0.935, Interference 0.826, Autonomy 0.862 for the maternal PBI at T2. The results showed that Cronbach’s alpha and McDonald’s omega were satisfactory.

### Construct validity of the Parental Bonding Instrument

The correlations between the scores of each PBI factor and the EPDS scores were determined to assess construct validity from Group 1 (Table 6). Each care factor was negatively correlated with EPDS scores, whereas each interference and each autonomy factor were positively correlated with EPDS scores.

## Discussion

To the best of the researchers’ knowledge, this is the first study to examine the reliability and validity of the PBI and the factor structure of each item in perinatal Japanese women.

In this study, it was decided to examine whether the items that satisfied the factor extraction value < 0.3 for all factors or ≥ 0.3 for multiple factors should be deleted in the EFA. It was found that items 7, 14, and 23 were deleted, and the EFA was performed again after deleting them, and a stable factor structure was obtained. A dataset not used in the first step was used to validate with the CFA of the three-factor model obtained from the EFA. All models had χ^2^/*df* ≤ 3, CFI > 0.95, SRMR ≤ 0.1, and RMSEA ≤ 0.08 (Table 5). Therefore, the value was close to a model hit.

In this EFA, the number of factors was determined by the scree plot method. At this stage, factor analysis was conducted assuming not only three factors, but also four factors, but it was not possible to obtain a stable factor structure with a four-factor structure in the present population. When confirmatory factor analysis was conducted using the previous four-factor structure^14^, for example, in the case of the prenatal father, the present factor structure provided a better fit. In addition, when compared with the previous two-factor structure^6^, the present factor structure also showed a better fit. This was also observed in other situations. Therefore, the three-factor structure obtained in the present study is appropriate for the factor structure of PBI for perinatal women (Tables 7, 8).

**Table 7:** Confirmatory factor models in the present study.

2-factor model (Parker et al.^6^)	4-factor model (Uji et al.^14^)	3-factor model based n EFA
Care: 1, 2, 4, 5, 6, 11, 12, 14, 16, 17, 18, 24	Care: 1, 5, 6, 11, 12, 17	Care: 1, 2, 4, 5, 6, 11, 12, 16, 17, 18, 24
Protection: 3, 7, 8, 9, 10, 13, 15, 19, 20, 21, 22, 23, 25	Indifference: 2, 4, 14, 16, 18, 24	Interference: 8, 9, 10, 13, 19, 20
	Over-protection: 8, 9, 10, 19, 20, 23	Autonomy: 3, 15, 21, 22, 25
	Autonomy: 3, 7, 15, 21, 22, 25

**Table 8:** Fit indices of different models.

	2-factor model (Parker et al.^6^)	4-factor model (Uji et al.^14^)	3-factor model based on EFA
CMIN/*df*	5.57	4.69	2.34
CFI	0.81	0.85	0.95
RMSEA	0.099	0.086	0.054
SRMR	0.105	0.098	0.068

χ^2^: Chi-squared; *df*: Degrees of freedom; CFI: Comparative fitness index; SRMR: Standardized root mean square residual; RMSEA: Root mean square error of approximation.

The reliability and validity of the PBI were examined in both pregnancy and the postpartum period. The results confirmed a three-factor structure of care, autonomy, and interference for the participants’ experiences of being parented. The items related to the participants’ fathers and those related to their mothers were separate. Reliability was high for all the factors assumed in the PBI items for both mothers and fathers. An examination of the correlation between the three PBI subscales and the EPDS scores showed a negative correlation between the father’s care and the EPDS scores and a positive correlation between the father’s interference and autonomy and the EPDS scores. In addition, whereas a negative correlation was found between the mother’s care and the EPDS scores, the autonomy of mothers and the interference of mothers were positively correlated with EPDS scores. This is consistent with the results of previous studies^18,19^ and demonstrates that the PBI has acceptable convergent validity. Therefore, the PBI can be considered a useful questionnaire that evaluates perinatal women’s experiences of being parented, that is, the parental attitudes that they acquired from their parents until 16 years of age.

In our study, three factors, Care, Interference, and Autonomy, were positively or negatively correlated. In addition, Interference and Autonomy were positively correlated with EPDS, whereas Care and EPDS were negatively correlated. In other words, lack of parental love, excessive parental interference, and low autonomy are associated with depression after childbirth.

According to developmental psychology so far, these three factors are closely related to each other. Adequate parental affection creates a sense of trust in others and in oneself, which in turn works to increase the child’s autonomy and self-esteem^21^. However, insufficient parental love creates mistrust. In addition, unhealthy parental love produces highly dependent children. This, in turn, reduces the autonomy and self-esteem of the child. On the other hand, parental behavior that attempts to overprotect the child, i.e., over-interference, inhibits the development of autonomy. Lack of parental affection, low autonomy, and low self-esteem have been reported to be associated with future depression^22–24^.

In light of these findings, it seems reasonable that Care and EPDS are negatively correlated and Autonomy and Interference and EPDS are positively correlated. In addition, Care, Interference, and Autonomy show a reciprocal relationship in the developmental stages, and it is also reasonable that there is a correlation between each factor.

In relation to the correlation coefficients between each factor, a negative correlation was found between care (for the care-related items, a higher score indicated a more loving parental attitude) and interference (for the interference-related items, a higher score indicated a more interfering parental attitude) and between care and autonomy (for the autonomy-related items, a lower score indicated that a greater value was placed on the child’s autonomy). On the other hand, interference and autonomy showed a positive correlation. This was the case for participants’ parental experiences in relation to both their fathers and mothers. This may be considered to be reasonable, because the more parents had caring feelings, the less interfering they were, and the more they valued the child’s autonomy. However, the more interfering a parent was, the less they valued the child’s autonomy. The negative correlation between care in fathers and mothers and EPDS scores and the positive correlation between interference and autonomy in fathers and mothers concur with the results of several previous studies that have shown that low care and high overprotection are risk factors for depression^18,19^.

There have been several previous studies of factor analysis of the PBI in women only, but the factor structure was different. A two-factor structure was obtained in studies of women who were neither perinatal nor child-rearing^16^, a three-factor structure was found in studies of postpartum women^17^, and a four-factor structure was found in studies of women raising kindergartners and elementary and middle school students^14,15^. Uji’s study conducted a factor analysis of the PBI among mothers raising elementary and middle school students in the same country as the present study and reported a four-factor structure (care, indifference, overprotection, and autonomy). The fourth factor, “indifference”, was raised as a factor that conflicts with “care”, which means coldness and aggression, as well as indifference. This factor arises from the projection of parental emotions toward a child who has been separated from his or her mother and child onto childhood experiences with their parents^14^. The subjects in the present study were in the “perinatal period,” a situation in which the distance between mother and child is close physically and psychologically, which may have made the emotions included in “indifference” less likely to arise. Similar to the present study, a past study confirmed the factor structure of the PBI in perinatal women, although that study was conducted in Spain, which is a different region and culture from Japan, and it confirmed the three-factor structure of the PBI found in the present study^17^. The names of the three factors that a past study confirmed differed from the names of factors confirmed in the present study, but no factors suggesting “indifference” occurred.

Therefore, this suggests that a factor structure with the same number of factors can be obtained if the subjects are women in the same life stage, even though the regions and cultures differ. In the factor analysis of the PBI for women, that is, not only national and regional differences, but also life stage factors such as perinatal and child-rearing, had a strong effect. The strength of this study was that it was possible to clarify the factor structure of the PBI at 25 weeks of gestation and at 1 month postpartum in a cohort with high homogeneity born in the same region. It was also confirmed that the PBI was reliable, validated, and had the same factor structure throughout the prenatal and postnatal periods. Compared with previous study of perinatal women^17^, the present study included a larger number of subjects, and the KMO measure of sampling adequacy at each time point confirmed a reasonable value for factor analysis. Unlike previous studies, in the present study, it was validated at two points, during pregnancy and postpartum, and the same factor structure was obtained, and a factor structure that can be used throughout the perinatal period was confirmed. In addition, the present study confirmed not only the reliability, but also the validity and factor validity by CFA. Thus, the present results provide a better factor structure of the PBI for use in perinatal women, which will allow researchers to examine the association between psychosocial factors and perinatal depression.

However, this study has various limitations. First, the participants in this study were recruited during prenatal classes for pregnant women. Participation in this study was optional. Consequently, the sample may have included a larger number of pregnant women interested in parenting. Thus, the sample may not have been representative of the general population. Second, because a self-administered questionnaire was used in this study, the presence or absence of psychopathology was not clarified through a structured interview or a similar measure. Therefore, it was not possible to exclude any effects that may have arisen from a participant’s mental illness. Finally, the present study was conducted at two time points: prenatal and postnatal. Therefore, it was not possible to use the test–retest method to test reliability between the two time points, which is done on the condition that the participants’ conditions have not changed.

## Conclusion

In this study, the reliability, validity, and factor structure of the PBI were examined in perinatal Japanese women at both pregnancy and postnatal time points. A three-factor structure of Care, Autonomy, and Interference was found for the fathers’ and mothers’ experiences of raising their children at both pregnancy and postnatal time points. The results showed high internal consistency and construct validity. In comparison with previous reports, the factor analysis of the PBI, especially for women, strongly reflected not only national and regional differences, but also life stage factors such as perinatal and child-rearing. It is hoped that the results obtained in this study will be used to more clearly examine the effects of parental parenting attitudes on perinatal depression.

## Methods

### Participants

This study was conducted between April 2006 and May 2020 at four hospitals, including Nagoya University Hospital and other hospitals cooperating with the research in Nagoya, Japan (with a population of approximately 2 million). Of the pregnant women who participated in a prenatal workshop, oral and written explanations of the purpose and methods of the study were given to those aged 20 years or older who could understand Japanese. Those who gave their written consent were analyzed. Having obtained their consent, the pregnant women were asked to fill out the Edinburgh Postnatal Depression Scale (EPDS) and the PBI questionnaires at 25 weeks of gestation (T1) and at 1 month postpartum (T2), and send their responses to the study group by mail.

### EPDS

The EPDS is a questionnaire created by Cox et al. for assessing postpartum depression^25^. A self-administered questionnaire consisting of 10 items and 4 test methods is used to evaluate each question item, with a score of 0–3 points. The total points range from 0 to 30 points. The EPDS has been used to assess both postpartum depression and depression during pregnancy^26^. The Japanese-language version of the EPDS showed excellent internal consistency (Cronbach’s alpha coefficient = 0.78) and retest reliability (Spearman’s correlation coefficient, r = 0.92)^27^. A score of 9 or more was assigned to screen for mild and major depressive episodes, with sensitivity of 75% and 82%, respectively, and specificity of 93% and 95%, respectively^27^.

### PBI

The PBI is a self-administered questionnaire that participants answer based on recollections of their experiences of being parented up to the age of 16 years. The scale consists of 25 items relating either to a participant’s father or mother. Parental attitude is evaluated on a 4-point scale (0 to 3 points) for 12 items relating to care and 13 items relating to overprotection. The care category is scored on a scale of 0–36, with higher scores indicating a more loving parental attitude and lower scores indicating an indifferent or rejecting parental attitude. The category of overprotection is scored on a scale of 0–39, with higher scores indicating overprotective or overly interfering parental attitudes and lower scores indicating a parental attitude that values spontaneity and child autonomy^6^. The Japanese-language version of the PBI that is used in Japan was translated and created by Kitamura and Suzuki, and its reliability and validity have been confirmed^11^.

### Statistical analysis

The participants were randomly divided into two groups (Group 1, n = 466 and Group 2, n = 466). Student’s *t* tests were conducted for age and EPDS scores compared between Group 1 and Group 2 (mean age, 32.73 ± 4.65 years; mean EPDS scores at T1, 5.09 ± 4.85; at T2, 5.13 ± 4.82). No significant differences were found between the two groups in age or EPDS scores at T1 or T2. There were some biases in the distribution of scores for some of the items. However, after examining the content of the items that showed a bias in the distribution of scores, all items were considered essential for measuring the concept of parenting experience. Therefore, not all items were excluded, and an EFA of all items of the PBI was conducted using data from Group 1. Because all factors were considered dependent on each other, the factor solution was sought after Promax rotation, which is an oblique rotation. The number of factors was determined by a scree plot. For items with a factor loading < 0.30 for all factors and for items with a factor loading > 0.30 for multiple factors, after deleting the items, EFA was performed again to confirm whether a stable factor structure could be obtained. In addition, models were created after deleting the items, and CFA was performed on each, and the model with the best fit was adopted. CFA was performed in Group 2 using the factor structure obtained above. These factor analyses were conducted after an assessment of specimen validity using the KMO Test^28^. A KMO value greater than 0.6 was considered to be appropriate for conducting the factor analysis. Chi-squared (χ^2^), degrees of freedom (*df*), comparative fitness index (CFI), standardized root mean square residual (SRMR), and root mean square error of approximation (RMSEA) were used to confirm the fitness of the data.

Results of 0 ≤  χ^2^/*df* ≤ 2, 0.97 ≤ CFI ≤ 1.00, 0 ≤ SRMR ≤ 0.05, and 0 ≤ RMSEA ≤ 0.05 indicated a good fit, and results of 2 <  χ^2^/*df* ≤ 3, 0.95 ≤ CFI < 0.97, 0.05 < SRMR ≤ 0.10, and 0.05 < RMSEA ≤ 0.08 indicated an acceptable fit^29^. Akaike’s information criterion was used when comparing models, and the model with the smallest value was considered the best model.

Using Group 1, reliability was confirmed by Cronbach’s alpha, Split-half, and Macdonald’s omega for each hypothesized factor. Construct validity was confirmed by calculating its correlation with the EPDS using Spearman’s correlation coefficient. Previous studies reported that depressive symptoms were associated with affectionless parental bonding^7,18,19^. This analysis provided evidence of construct validity.

All statistical analyses were conducted using SPSS version 26.0 and Amos 27.0 (IBM Japan, Tokyo, Japan).

### Ethics statement

The study was explained to all participants both verbally and in writing, and written, informed consent was obtained from each participant. This study protocol was approved by the Ethics Committee of the Nagoya University Graduate School of Medicine. The study was conducted in accordance with the established ethical standards of all institutions.
